# Comparing Different Approaches for Subtyping Children with Conduct Problems: Callous-Unemotional Traits Only Versus the Multidimensional Psychopathy Construct

**DOI:** 10.1007/s10862-018-9653-y

**Published:** 2018-03-09

**Authors:** Olivier F. Colins, Henrik Andershed, Randall T. Salekin, Kostas A. Fanti

**Affiliations:** 1 Department of Child and Adolescent Psychiatry, Curium-Leiden University Medical Center, Endegeesterstraatweg 27, AK 2342 Leiden, The Netherlands; 2 Center for Criminological and Psychosocial Research, Örebro University, Örebro, Sweden; 3 Department of Psychology, University of Alabama, Tuscaloosa, USA; 4 Department of Psychology, University of Cyprus, Nicosia, Cyprus

**Keywords:** Antisocial process screening device, Inventory of callous-unemotional traits, Psychopathy, Conduct disorder, Limited prosocial emotions

## Abstract

The aim of this study was to compare two youth psychopathy models (i.e., callous-unemotional versus multidimensional model) in their ability to predict future and stable conduct problems (CP). At baseline, mothers and fathers of 321 boys and 369 girls (ages 7–12) completed measures that tap callous-unemotional and other psychopathic traits. Parent-reported CP was collected at baseline and at 6- and 12 month follow-ups. Children were assigned to mutually exclusive groups based on their levels of CP and psychopathic traits. Children with CP who manifested callous-unemotional traits (*Callous-Unemotional + CP*) were occasionally at risk for future and stable CP. Yet, across gender, children with CP scoring high on all psychopathic trait dimensions (*Psychopathic Personality + CP*) showed the most robust and highest risk for future and stable CP. Also, *Callous-Unemotional + CP* children, and children who were only high in CP, often were at similar risk for future CP. The findings suggest that the callous-unemotional model is less sufficient than the multidimensional model in predicting future and stable CP. This can be concluded for both boys and girls and calls for more research reconsidering the multidimensional nature of psychopathy for CP subtyping purposes.

**Electronic supplementary material:**

The online version of this article (10.1007/s10862-018-9653-y) contains supplementary material, which is available to authorized users.

## Introduction

Children and adolescents with conduct problems (CP) constitute a heterogeneous group, not only in the types of CP they exhibit (e.g., Lindhiem et al. 2015), but also in their risk for future antisocial outcomes (e.g., Odgers et al. 2008). Past research showed that callous-unemotional (CU) traits help to identify a subgroup of children with CP who exhibit a more severe and stable pattern of CP compared to youths with CP low on CU traits (for a review see: Frick et al. 2014). Reflecting this body of evidence, CU traits have become increasingly included in theoretical models^1^ and empirical studies on CP, and will influence clinical work with children and adolescents since *DSM-5* already incorporated a CU-based specifier for the diagnosis of conduct disorder (APA 2013; Salekin 2016). Regardless of the relevance of studying CU traits in relation to CP, several critical questions remain: Are CU traits enough to identify youth with the most severe conduct problems? Are CU traits the best predictor of future and stable conduct problems? Do we need greater representation of psychopathic traits? And, are psychopathic traits without CP worth consideration for classifying youth?

### Are CU Traits Enough to Identify Children with the most Severe Conduct Problems?

CU traits represent a downward extension of the affective features of adult psychopathy and are commonly characterized by deficient empathy and guilt, insensitivity to others’ feelings, and shallow emotions. Importantly, psychopathy is commonly described as a constellation of several *co-occurring traits* that load onto various dimensions other than CU traits as well, including interpersonal, behavior/lifestyle, and antisocial dimensions (Cooke and Michie 2001; Frick et al. 2000; Hare and Neumann 2008). Notwithstanding that CU traits only capture one of the psychopathic trait dimensions, there is a tendency to use CU traits interchangeably with psychopathy, especially when CU traits co-occur with CP (e.g., Breeden et al. 2015; Jones et al. 2009). Also, it has been argued that children and adolescents with CU traits often show the highest levels of other psychopathic traits, and that using only CU traits, therefore, often designates similar groups of youth with CP as when using composite scores on all psychopathic trait dimensions (e.g., Frick 2009). This expectation can be challenged for at least two reasons. First, youth who are only high on CU traits can be differentiated from those who are high on all psychopathic trait dimensions, with the latter group displaying the highest levels of *concurrent* or *past* CP, aggression and antisocial behavior (e.g., Christian et al. 1997; Colins et al. 2012; Frick and Hare 2001). Second, prior work has shown that the interaction effect between the three psychopathic traits dimensions exhibits a stronger relation with *concurrent* or *past* CP (Colins et al. 2014) and proactive aggression (Orue and Andershed 2015) than any psychopathic trait dimension on its own (main effect). In sum, there is evidence to suggest that when classifying individuals on all psychopathic trait dimensions a more severe subgroup of children with CP can be identified.

### Are CU Traits the Best Predictor of Future and Stable Conduct Problems?

CU traits are viewed to be important for designating *within* children and adolescents with CP a group who is at risk for *future* severe and stable CP (e.g., Frick et al. 2014). Various longitudinal studies revealed prospective relations between CU traits and future CP, aggression, and delinquency. Yet, most of these studies assessed or studied CU traits without reference to the other psychopathic traits dimensions. Such a methodology cannot provide information as to whether it is the CU or the other psychopathic trait dimensions that account for the results (e.g., Frick et al. 2014), which is unfortunate in the light of evidence that interpersonal and behavioral/lifestyle trait dimensions are uniquely positively related to future CP, aggression, and criminality, whereas CU traits are not, or less strongly so (e.g., Colins et al. 2015a, b; Munoz and Frick 2007). Also, studies focusing on CU traits in children and adolescents with CP often include interpersonal traits in their measure of CU traits (e.g., Barker et al. 2011; Rowe et al. 2010), which raises the intriguing question to what extent the association between CU traits and CP identified by prior work might be due to the shared variance with the interpersonal traits dimension. In sum, CU traits may not be the sole or even the strongest predictor of future CP and taking other psychopathic traits into account may be important for identifying high risk children.

### Do we Need a Greater Representation of Psychopathic Traits Other than CU?

Addressing the aforementioned issues is particularly relevant as many researchers link their studies to adult psychopathy whilst only assessing CU traits and CP (e.g., Dadds et al. 2014; De Brito et al. 2009). Several researchers are reluctant to equate CU traits with psychopathy, and prefer using the broader, multidimensional construct of psychopathy to identify juveniles with a personality that at least at the surface resembles adult psychopathy (e.g., Salekin 2016). Scholars also suggested that future revisions of *DSM* and *ICD* should consider to have a conduct disorder diagnosis with three specifiers (interpersonal, CU, and behavioral/lifestyle) to enhance diagnostic information and treatment planning (Salekin 2017). Prior work that assigned individuals to mutually exclusive groups based on their CP and CU scores already showed that CP youth with CU traits are the worst in terms of concurrent and future antisocial behavior (e.g., Fanti and Kimonis 2012; Rowe et al. 2010). Without disregarding the likelihood that CU, on its own, may have prognostic usefulness, children with CP who are high on *all* psychopathic trait dimensions might even be worse in concurrent and future CP.

### What about Psychopathic Traits that Occur in Children without Concurrent CP?

The role of the antisocial dimension, which primarily indexes criminal behavior, is still debated in the adult psychopathy literature. Various researchers argue that this dimension should not be considered, for example, because it introduces a prognostic tautology in predicting future criminality (e.g., Cooke et al. 2007). This concern is supported by work showing that the relation between psychopathy and future criminality is largely driven by this antisocial dimension (e.g., Walters et al. 2008), though it must be noted that this is not always the case (e.g., Colins et al. 2015a; Vitacco et al. 2005). Of note, most assessment tools that are commonly used in contemporary research on childhood and adolescent psychopathy do not include an antisocial dimension.^2^ However, as CP are related to past criminality (e.g., Colins 2016) and constitute a risk factor for future criminality (e.g., Babinski et al. 1999), one could argue that CP serve as a proxy for criminality as indexed in Hare’s (Hare 2003) adult psychopathy conceptualization (Skeem et al. 2011). If so, CP can be used to empirically test if being high in CU traits or being high on all psychopathy dimensions is also predictive of future CP in children *without* CP. The outcome of such test is important because CU traits that occur in the absence of CP or other forms of antisocial behavior are considered to bear clinical significance (Viding and McCrory, 2012).

## The Current Study

The present study will test the usefulness of using one (CU) or three psychopathic trait dimensions for identifying CP children at risk for future and stable CP. This study also seeks to clarify if both models are predictive of future and stable CP, without the co-occurrence of high baseline levels of CP. To enable comparison with prior work that triggered the focus on CU traits, this study will assess psychopathic trait dimensions by means of the Antisocial Process Screening Device (APSD; Frick and Hare 2001) and the Inventory of Callous-Unemotional Traits (ICU; Frick 2004). Information from parents were combined by applying the commonly applied highest score prevail method to combine APSD and ICU ratings from fathers and mothers (e.g., Frick et al. 2000). Finally, for consistency with current clinical practice (Cogill and Sonagu-Barke 2012), this study will assign children to mutually exclusive groups in an effort to provide clinical information for psychopathic traits-based specifiers in relation to conduct problems.

### Hypotheses

First, it was expected that children could be assigned to at least six groups: (1) low CP and low on all psychopathyic trait dimensions (*Control*); (2) high CP and low on all psychopathyic trait dimensions (*CP Only*); (3) low CP and high on CU only (*CU Only*); (4) low CP and high on all psychopathic trait dimensions (*Psychopathic Personality Only*); (5) high CP and high on CU only (*CU + CP*); and (6) high CP and high on all psychopathic trait dimensions (*Psychopathic Personality + CP*). Second, mirroring the expectation that children with CP who manifest psychopathic traits constitute a severe CP subgroup (e.g., Frick et al. 2014; APA 2013), it was hypothesized that *Psychopathic Personality + CP* and *CU + CP* children will be higher in baseline CP than children in the other groups. Crucially, it was also expected that the *Psychopathic Personality + CP* children will be higher in baseline CP than *CU + CP* children. Third, it was expected that *Psychopathic Personality + CP* and *CU + CP* children will be at a higher risk for future and stable CP than their *CP Only* and *CU Only* counterparts, though *Psychopathic Personality + CP* children were expected to be at the highest risk. Of note, boys are at higher risk for CP and psychopathic traits than girls (e.g., Colins et al. 2014; Fanti, 2013; Fanti and Kimonis 2012), and within gender cut-offs have been used in prior CU and psychopathy research to reduce the risk of gender bias (e.g., Colins et al. 2016; Sprague and Verona, 2010, Dadds et al. 2009). Therefore, we will account for gender differences in levels of CP and psychopathic traits, by testing the aforementioned hypotheses separately for boys and girls. Doing so will also enhance comparison with other work included in this special issue (see Frogner, Andershed, & Andershed, *this volume*).

## Method

### Participants and Procedure

Following study approval by the Cyprus Ministry of Education and Bioethics Committee and school boards of all participating schools, 26 schools (10 from rural areas) in the four school districts in Cyprus were randomly selected to ensure that the sample was representative of the population. Before data collection, signed parental consent and youth assent were obtained from all participating families (85% of parents and children agreed to participate). Children were given a sealed envelope that included the questionnaires to be completed by both parents. Parents were instructed to place the completed questionnaires in the sealed envelope and return them to the child’s school. This procedure resulted in responses from parents from 1701 children (806 boys and 895 girls) at study commencement. Next, the four 6-year old and ten 13-year old children were not included in the present study. Finally, only the children with complete data from both parents on the main study variables across the three waves of data collections were included in the present study (details available upon request). This strict procedure was followed to use the same sample of boys and girls for all analyses (i.e., using different samples would have influenced the z-scores based group assignments and subsequent analyses).

The final sample used in the present study consisted of 690 families (321 boys and 369 girls) in Cyprus at three time points, 6-months apart. Children ranged in age from 7 to 12 (*M* = 8.84 years, *SD* = 1.47) at study commencement. The sample was evenly divided in the first through fifth elementary grades with approximately 16–20% of the sample in each grade. Only 6% of the sample was in grade 6. The sample was diverse in terms of parental educational levels: 11.7% of the parents did not complete high school, 37.9% had a high school education, and 50.4% had a university degree, which is representative of the population in Cyprus (Ministry of Finance, Statistical Service, 2012).

### Measures

#### Callous-Unemotional Traits

CU traits were assessed with the 24-item ICU- parent version*.* ICU items were rated on a 4-point Likert scale (0 = “not at all” to 3 = “definitely true”), with higher scores indicating greater CU traits. Mother and father ICU total scores were highly correlated (*r* = .68).

Grandiose and impulsive traits were measured with the APSD-parent version. The items are scored on a 3-point scale (0 = “not at all true,” 1 = “sometimes true,” and 2 = “definitely true”). Only the 7-item Grandiose (cf. interpersonal traits dimension) and 5-item Impulsivity (cf. behavioral/lifestyle traits dimension) subscales were used, based on the availability of the CU measure of the ICU, which was developed to enable a more comprehensive assessment of CU traits than the APSD (e.g., Frick 2009). Mother and father reports for both scales were highly correlated (*r* = .69–.72).

#### Conduct Problems

CP were measured with the Child Symptom Inventory for Parents-4 (CSI-4; Gadow and Sprafkin 2002), which assesses the frequency of 15 symptoms of CD on a 4-point scale (ranging from 0 = “never” to 3 = “very often”). The CSI-4 was administered at three time points. Mother and father reports were highly correlated at each time point of measurement (*r*_*range*_ = .70–.75). Stable CP was calculated through identifying those .5 SD above mean in CP within each gender at both wave 2 and 3. All other children were placed in the no-stable conduct problem category.

### Data-Analyses

First, mother- and father-reported CP, ICU, and APSD scores were combined by taking the higher rating between parents at the item level, a strategy that is commonly used in childhood CU and psychopathy research (e.g., Frick et al. 2000; Colins, Fanti, Larsson, & Andershed), and is supported by research showing moderate to strong correlations between parents reports about their children’s behavior (e.g., Achenbach et al. 1987; Daveé, et al. 2008). The combined total scores demonstrated good internal consistency: CU (α = .86), Grandiose (α = .70), Impulsivity (α = .73), and CP (α across time = .85 to .89). Second, Pearson product moment correlations were first estimated in boys (*n* = 321) and girls (*n* = 369) to examine the bivariate relationships among the study variables. Third, a cutoff of .5 *SD* above the mean was used to dichotomize children into high (above cutoff) and low levels (below cut-off) of baseline CP, Grandiosity, CU, and Impulsivity. This cut-off was chosen to enable comparison with prior work that used distribution based cut-off scores (SD, median splits and percentiles) to assign children to high CU or high psychopathic traits groups (e.g., Pasalich et al. 2012; Schwenk et al. 2012; Van Baardewijk et al. 2009; Viding et al. 2008), but also to assure that enough children would be assigned to the groups of interest and thus to optimize the power for testing competing models. To account for gender differences in levels of CP and psychopathic traits, and in line with prior work (e.g., Dadds et al. 2009), these group assignments and z-score transformations were performed separately for boys (*n* = 321) and girls (*n* = 369). Based on being above or below the cut-off, children were assigned to the aforementioned six mutually exclusive groups (see also Table 1). Following the study’s aims, children being assigned to other groups (e.g., only high on grandiosity or impulsivity) were not included in the remaining analyses. Fourth, ANOVA analyses were performed to test for group differences in sociodemographic characteristics and CP at baseline with Bonferroni correction or with Games Howell correction in case the homogeneity of variance assumption was violated. Fifth, five dummy coded group variables (*CP Only*, *CU Only*, *Psychopathic Personality Only*, *CU + CP; and Psychopathic Personality + CP*) were *simultaneously* entered as independent variables into linear regressions predicting future CP at 6- and 12-months follow-up, and into logistic regressions predicting the binary measure of stable CP. Sixth, to check the robustness of our findings, we also applied the .75 *SD* above the mean as a cut-off to assign boys and girls to mutually exclusive groups (see Supplementary Material for descriptive information), and repeated all the analyses described earlier with this alternative cut-off.^3^ Statistical analyses were performed by means of SPSS 23.0, and we used *p* < .05 as the indicator of statistical significance.

**Table 1:** Correlations between study variables for the Boys (*n* = 321) and Girls (*n* = 369)

	(1)	(2)	(3)	(4)	(5)	(6)	(7)	(8)	(9)	Descriptive	Descriptive
										Boys	Girls
Parental SES (1) ^b^	–	−.02	−.10	−.13*	−.27***	−.09	−.10	−.05	−.05	4.43 (1.23)	4.48 (1.22)
Age (2)	−.03	–	−.12*	−.06	−.02	.01	−.08	−.19***	−.004	8.72 (1.46)	8.95 (1.48)
CP Baseline (3)	−.06	.02	–	.49***	.45***	.51***	.56***	.49***	.35***	1.49 (1.99)	1.00 (1.84)
Grandiosity (4)	−.09	−.02	.41***	–	.58***	.63***	.35***	.31***	.22***	4.49 (3.16)	3.98 (3.07)
CU (5)	−.19***	.01	.39***	.51***	–	.58***	.30***	.27***	.13*	22.47 (10.79)	21.10 (10.44)
Impulsivity (6)	−.02	.01	.52***	.62***	.58***	–	.41***	.30***	.20***	4.91 (3.09)	3.99 (2.64)
CP Year 2 (7)	.04	−.07	.44***	.30***	.30***	.36***	–	.60***	.60***	1.28 (1.70)	0.93 (1.76)
CP Year 3 (8)	.07	−.03	.49***	.36***	.29***	.39***	.70***	–	.60***	1.40 (2.06)	0.96 (1.81)
Stable CP (9)	.05	−.07	.46***	.32***	.26***	.40***	.72***	.78***	–	30 (9.3%)^a^	49 (13.3%)^a^

Correlation coefficients for boys and girls are above and below the diagonal, respectively; *CP*, conduct problems; *CU*, callous-unemotional

^a^ N and % for Stable CP

^b^Based on parental education (1 = elementary school; 2 = middle school; 3 = technical school; 4 = high school; 5 = college; 6 = university)

* p < .05; ** *p* < .01; *** *p* < .001

## Results

### Descriptive Information

Descriptive information for and correlations between all variables can be found in Table 1. As shown in the Table, all psychopathic trait dimensions were significantly positively related to each other and to baseline CP across gender. Baseline CP and the psychopathic trait dimensions were positively related to future and stable CP. Because age and parental SES were occasionally related to the psychopathic trait dimensions and future CP, they were included in the regression analyses as covariates.

### Baseline Differences between the Groups

#### Boys

Table 2 shows that *CP Only*, *CU + CP,* and *Psychopathic Personality + CP* boys were not significantly different from each other in terms of CP, although they all scored higher than boys in the other three groups. These findings were replicated when using an alternative cut-off for assigning boys to mutually exclusive groups (see Table 3).

**Table 2:** Group comparisons of baseline levels of conduct problems and psychopathic traits among Boys (*n* = 236) and Girls (*n* = 269) when using .5 SD as Cut-Off

		Control	CP only	CU only	PP only	CU + CP	PP + CP	Group comparisons
		(1)	(2)	(3)	(4)	(5)	(6)	Within gender*
Boys^a^	Conduct problems	0.41 (0.63)	3.58 (1.00)	0.70 (0.70)	0.93 (0.83)	3.50 (0.85)	5.26 (3.06)	2 5 6 > 1 3 4
	Grandiosity	2.48 (1.68)	4.08 (1.31)	3.52 (1.83)	10.28 (2.61)	4.30 (1.34)	9.78 (2.02)	2 3 4 5 6 > 1; 4 6 > 2 3 5
	CU	14.99 (7.12)	20.83 (6.19)	33.09 (3.87)	36.36 (5.40)	35.40 (4.69)	36.17 (7.63)	3 4 5 6 > 12
	Impulsivity	2.85 (1.80)	3.91 (1.93)	3.74 (1.60)	9.21 (2.08)	5.00 (1.05)	10.04 (1.82)	2 3 4 5 6 > 1; 4 6 > 2 3 5
Girls^b^	Conduct problems	0.18 (0.39)	2.78 (1.57)	0.50 (0.51)	0.64 (0.50)	2.61 (0.77)	5.21 (3.99)	2 5 6 > 1 3 4; 6 > 5; 4 > 1
	Grandiosity	2.10 (1.60)	2.55 (0.52)	2.69 (1.04)	9.71 (2.76)	3.38 (1.32)	8.88 (2.15)	4 5 6 > 1; 4 6 > 2 3 5
	CU	14.03 (6.41)	15.00 (6.26)	31.38 (4.84)	34.78 (5.22)	33.08 (4.48)	43.71 (5.97)	3 4 5 6 > 1 2
	Impulsivity	2.30 (1.58)	3.89 (1.27)	3.73 (1.37)	8.14 (1.56)	3.92 (1.11)	8.88 (1.89)	2 3 4 5 6 > 1; 4 6 > 2 3 5

*CP*, conduct problems; *CU*, callous-unemotional; *PP*, Psychopathic Personality; Group comparisons for underlined and non-underlined variables are based on Bonferroni and Games Howell post-hoc tests, respectively;

^a^Within boys, the number (%) assigned to each group was: (1) = 154 (63.3%); (2) = 12 (5.1%); (3) = 23 (9.7%); (4) = 14 (5.9%); (5) = 10 (4.2%), and (6) = 23 (9.7%);

^b^Within girls, the number (%) assigned to each group was: (1) = 183 (68.0%); (2) = 9 (3.3%); (3) = 26 (9.7%); (4) = 14 (5.9%); (5) = 13 (4.8%), and (6) = 24 (8.9%);

**Table 3:** Group comparison on baseline levels of conduct problems and psychopathic traits among Boys (*n* = 248) and Girls (*n* = 284) when using .75 SD as Cut-Off

		Control	CP Only	CU Only	PP Only	CU + CP	PP + CP	Group comparisons
		(1)	(2)	(3)	(4)	(5)	(6)	Within gender
Boys	Conduct problems	0.49 (0.69)	3.53 (0.97)	0.70 (0.73)	0.90 (0.87)	3.78 (1.05)	5.35 (3.16)	2 5 6 > 1 3 4
	Grandiosity	2.61 (1.74)	4.23 (1.36)	4.15 (1.69)	11.0 (2.75)	4.21 (1.25)	9.82 (2.16)	2 3 4 5 6 > 1; 4 6 > 2 3 5
	CU	15.89 (7.48)	21.46 (6.34)	34.35 (3.20)	37.80 (4.16)	35.50 (3.76)	37.47 (7.58)	3 4 5 6 > 1 2
	Impulsivity	3.20 (2.05)	4.08 (1.93)	4.35 (1.95)	10.0 (1.94)	5.64 (1.34)	10.18 (1.67)	4 5 6 > 1; 4 6 > 2 3 5
Girls	Conduct problems	0.29 (0.55)	4.50 (1.29)	0.78 (0.79)	0.94 (0.75)	4.00 (1.73)	6.47 (4.55)	2 3 4 5 6 > 1; 2 6 > 3 4
	Grandiosity	2.39 (1.76)	2.25 (1.50)	3.59 (1.79)	9.70 (2.52)	4.20 (1.30)	9.60 (2.06)	3 4 6 > 1; 4 6 > 2 3 5
	CU	14.84 (6.88)	23.50 (7.72)	34.72 (4.79)	34.94 (4.59)	34.00 (3.94)	35.67 (6.08)	3 4 5 6 > 1
	Impulsivity	2.52 (1.65)	4.00 (0.82)	3.78 (1.31)	8.00 (1.54)	4.60 (0.55)	9.20 (2.18)	2 3 4 5 6 > 1; 4 6 > 2 3 5

*CP,* conduct problems; *CU*, callous-unemotional; *PP*, Psychopathic Personality; Group comparisons for underlined and non-underlined variables are based on Bonferroni and Games Howell post-hoc tests, respectively;

#### Girls

The group differences for girls were similar to those reported for the boys, though it must be noted that *Psychopathy Only* girls were significantly higher in CP than girls in the *Control group*, whereas *Psychopathic Personality + CP* girls had significantly more CP than *CU + CP* girls (Table 2). These latter differences disappeared when using the .75 *SD* cut-off (see Table 3).

### Predicting Future and Stable Conduct Problems

#### Boys

Table 4 shows that *Psychopathic Personality + CP* was the strongest predictor for CP, followed by *CP Only* boys. The other groups were not significantly at risk for future CP, except that *CU + CP* boys were significantly predictive of CP at 12-month follow-up. *Psychopathic Personality + CP* and *CP Only* boys were the only boys who were at risk for stable CP. Similar findings were revealed when using the .75 *SD* cut-off (see Table 5).

**Table 4:** Predicting future and stable conduct problem among boys (n = 321) and Girls (n = 369) when using .5 SD as Cut-Off

	6-Months later				12 Months later				Stable			
	Boys		Girls		Boys		Girls		Boys		Girls	
	*β*	*p*	*β*	*p*	*β*	*p*	*β*	*p*	*OR*	*p*	*OR*	*p*
CP only	.17**	.001	*.*10*	<.05	.14**	.008	.04	.37	5.52***	.02	5.08*	.03
CU only	−.04	.48	.05	.33	−.02	.70	.02	.65	>0.00	.99	1.69	.42
Psychopathic personality only	.08	.13	.12*	.02	.09	.07	.11*	.02	2.43	.27	5.64**	.008
CU + CP	.07	.19	.18***	<.001	.12*	.02	.14**	.004	1.40	.76	4.22*	<.05
Psychopathic personality + CP	.30***	<.001	.38***	<.001	.34***	<.001	.46***	<.001	9.65***	<.001	17.42***	<.001

*β*, standardized beta; *OR*, Odds Ratio; *CP*, conduct problems; In all analyses age and parental SES were included as control variables. Confidence intervals for unstandardized betas and *OR* are presented in the Supplementary Material

* *p* < .05; ** *p* < .01; *** *p* < .001

**Table 5:** Predicting future and stable conduct problem among Boys (n = 248) and Girls (n = 284) when using .75 SD as Cut-Off

	6-Months later				12 Months later				Stable			
	Boys		Girls		Boys		Girls		Boys		Girls	
	*β*	*p*	*β*	*p*	*β*	*p*	*β*	*p*	*OR*	*p*	*OR*	*p*
CP only	.15**	.005	.11*	.03	.12*	.02	.06	.19	4.04*	.05	11.03*	.02
CU only	−.05	.34	.08	.10	−.06	.30	.04	.44	>00	.99	1.49	.49
Psychopathic personality only	.02	.72	.13**	.01	.05	.38	.10**	.04	1.36	.78	3.65*	.04
CU + CP	.10	.07	.13**	.007	.21***	<.001	.05	.30	.78	.82	8.23*	.03
Psychopathic personality + CP	.20***	<.001	.32***	<.001	.24***	<.001	.44***	<.001	6.54**	.001	18.86***	<.001

*β*, standardized beta; *OR*, Odds Ratio; *CP*, conduct problems; In all analyses age and parental SES were included as control variables;

* p < .05; ** p < .01; *** p < .001

#### Girls

*Psychopathic Personality + CP* girls were at the highest risk for CP at 6- and 12-months, followed by *CU + CP* and *Psychopathic Personality Only* girls. *CP Only* girls were at risk for CP at the 6- but not at the 12-month follow-up (Table 4). *Psychopathic Personality + CP girls* were at the highest risk for stable CP, followed by *Psychopathic Personality Only*, *CP Only*, and *CU + CP* girls (Table 4). These findings were replicated whilst using the alternative cut-off, except that *CU + CP* was no longer predictive of CP at 12-month follow-up (see Table 5).

## Discussion

The present study showed that the most robust and strongest prospective relation with future and stable CP occurred when high levels of interpersonal (grandiosity), CU, *and* behavioral/lifestyle traits co-occurred with high levels of CP at baseline (*Psychopathic Personality + CP*). In children *without* CP, being high on CU traits only (*CU Only*) and being high on all psychopathic traits dimensions (*Psychopathic Personality Only*) was occasionally, though only weakly, predictive of future and stable CP. Although the CU-based subtyping of children with CP received some support, current results also suggest that using the multidimensional construct of psychopathy is more useful to identify children at increased risk for future and stable CP. Of note, the number of boys and girls assigned the *CP Only* group were quite low, suggesting that CP quite seldom comes along without personality traits than can be tied to psychopathy.

Following prior work (e.g., Fanti, 2013), boys and girls could be assigned to *CP Only* and *CU + CP* groups. Importantly, across gender, children with CP who were high on all three psychopathic traits dimensions could also be identified (i.e., *Psychopathic Personality + CP*), providing evidence for additional heterogeneity in CP. Whereas the *CP Only* and *CU + CP* groups showed similar levels of CP at baseline, higher levels of CP were revealed in the *Psychopathic Personality + CP* than in the *CP Only* and *CU + CP* groups, though these differences were most often not statistically significant after correcting for multiple group comparisons. Our findings suggest that when high levels of CU traits do not co-occur with high levels of interpersonal (e.g., grandiosity) and behavior/lifestyle psychopathic traits, CU traits are not associated with CP. These findings challenge the view that being high on CU traits and being high on all three psychopathy dimensions identifies largely overlapping groups of children with CP (See Introduction: Question 1), and underscore the relevance of systematically reporting how each psychopathic trait dimension is uniquely related to variables of interest (See Introduction: Question 2).

CU traits also have been considered important for predicting future and stable CP. Notwithstanding that *CU + CP* children, especially girls, were at risk for future and stable CP, *CP Only* children were often equally, and sometimes even at a higher risk than *CU + CP* children. These findings contradict the expectation that using CU traits will identify a subgroup of children with CP who are at higher risk for future and stable CP. Interestingly, *Psychopathic Personality + CP* children consistently were at risk for future and stable CP at 6- and 12-months, across gender, and when using the two alternative cut offs (.5 and .75 *SD*). This finding dovetails well with evidence that the combination of psychopathic traits dimension is most strongly related to concurrent CP and aggression (Colins et al. 2014; Orue and Andershed 2015), confer the greatest risk for future antisocial behavior (Fanti and Kimonis 2012), and are associated with stability of CP (Klingzell et al. 2016). Altogether, our findings provide preliminary support for the suggestion (e.g., Salekin 2016, 2017) to include a greater representation of psychopathic traits dimensions for subtyping children with CP and conduct disorder (See Introduction: Question 3).

Consistent with prior work showing that past antisocial behavior is the best predictor of future antisocial behavior (e.g., Colins et al. 2015b), children with high levels of CP (i.e. *CP Only, CU + CP; Psychopathic Personality + CP*) were generally at increased risk for future and stable CP. Because the *Psychopathic Personality + CP* children were higher in baseline levels of CP, it may come as no surprise to see that these children were at higher risk to engage in future CP. This higher level of baseline CP cannot be the sole explanation, though, for at least two reasons. First, while *CP Only* and *CU + CP* boys were not different in baseline levels of CP, *CP Only* boys were repeatedly at risk for CP, whereas *CU + CP* boys showed higher risk only at 12 month follow-up. If baseline CP would entirely drive the relation with the outcomes, then one would expect to find quite robust prospective relations among *CU + CP* boys and later CP as well. Although this set of findings provides some evidence for the importance of CU traits for subtyping purposes among CP children, it also indicates that when using all psychopathic traits dimensions, children who are assumed to be less severe (i.e., *CP Only*) are as likely as *CU + CP* children to engage in CP. Second, in girls *without* CP, those high on all psychopathic traits dimensions (*Psychopathic Personality Only*) were at increased risk for future and stable CP, whereas those high on CU traits (*CU Only*) were not. Thus, even without introducing a prognostic tautology (See Introduction: Critical Issue 4), being high on three psychopathic traits dimensions is predictive of future and stable CP, a finding that again underscores the relevance to study psychopathy as a constellation of *co-occurring* traits.

Because the power of the test of the difference between two regression coefficients is quite low, especially with groups with small numbers as was the case in the present study (e.g. Kenny, 1987), we did not test if the strength of the association between group assignment and future and stable CP was significantly different across gender. However, the results from the present study do suggest that it may be important to take gender into account in future research on the topic. This is because high levels of psychopathic traits that did not occur with CP (*CU Only* and *Psychopathic Personality Only*) were only predictive of future and stable CP in girls. Likewise, high CU levels that co-occurred with high levels of CP (*CU + CP*) were mostly prospectively related to CP outcomes in girls, This latter finding suggests that CU-based specifiers for CP subtyping purposes, such as the DSM-5 specifier, might be more useful for girls than for boys.

The strengths of the present study include the longitudinal design, the multi-informant approach and the use of measures (APSD and ICU) and constructs (CP) that have been extensively used in prior work. The results of the study should be interpreted in the light of several limitations. First, using 1.00 *SD* above the mean as cut off resulted in groups with too few boys and girls to directly compare the models (groups) of interest with each other. However, various approaches have been used by prior work to identify children with CP who also have high levels of CU, including median split and percentile-based cut-off scores (e.g, Klapwijk et al. 2016; Pasalich et al. 2012; Schwenk et al. 2012). Therefore, future studies using other cut-off scores, and/or methods to differentiate within children with CP, are warranted. Second, the main aim was to compare two competing approaches to identify children with a personality that at least at the surface looks like adult psychopathy. As such, for comparison purposes we did not include other groups (e.g., CP youth high on grandiose traits only) that can be tied to recently proposed, alternative specifiers for CD (Salekin 2016). Third, it can be argued that our analytical approach (categorizing continuous variables) was based on arbitrary cut-offs and may result in loss of statistical power and increased probability of committing type-I errors (e.g., MacCallum et al. 2002). We acknowledge these arguments but also note that despite this assumed reduction in terms of statistical power we nevertheless found prospective relations between group-membership categories and future and stable CP. Both continuous and categorical approaches are useful and necessary (see for example also Farrington and Loeber, 2000; Lilienfeld 2014). Fourth, future and stable CP are an important albeit not the only outcome of interest that can be used to test the clinical meaningfulness and prognosis of the group assignments. Future research should use other (e.g., substance use) or more fine-grained CP-related baseline and outcome variables (e.g., aggression). Fifth, some comparison groups were small in number, a feature that may affect the validity of our findings and limit the likelihood to find statistically significant group differences. Studies with larger sample sizes, therefore, are an important avenue for future research that uses the analytical approach reported in the current paper. Finally, prior work on CP and/or psychopathic traits showed that findings stemming from research with community samples cannot always be replicated among clinic-referred and criminal justice-involved samples (e.g., Colins et al. 2011; Colins 2016; Jambroes et al. 2016), and that the prognostic usefulness of psychopathic traits may reduce as follow-up time increases (e.g., Cauffman et al. 2009). Therefore, studies that use data from clinic-referred or criminal justice-involved CP youth and a > 12 months follow-up interval, are highly relevant when trying to replicate the current study’s findings.

If replicated, the present study provides important practical and theoretical information. First, it suggests that alternative specifiers for future versions of the *DSM* or *ICD* must be considered (Salekin 2016, 2017). Second, the construct of adult psychopathic personality has been extended to childhood as a multidimensional construct (e.g., Christian et al. 1997). Our findings showed that increasing the representation of the condition may help identify those who are at risk for long term CP and even psychopathy in the future. Third, the current study suggests that *CU + CP* boys are also at increased risk for future and stable CP, though to a lesser extent than *Psychopathic Personality + CP* boys limiting its predictive potential. Thus, identifying a subtype of children showing a constellation of psychopathic traits can inform future clinical work and provide evidence for a group of children at greater need for intervention. Finally, the present study’s findings suggest that future research should test the prognostic usefulness of different psychopathic trait-based subtyping approaches separately in boys and girls.

## Electronic supplementary material

ESM 1 (DOCX 36 kb)

## Footnotes

1 For example, there is a theory stating that children who are less sensitive to punishment have difficulties to develop adequate forms of guilt and empathy, will develop CU traits, and eventually display severe CP (e.g., Frick and Viding, 2009).

2 The Psychopathy Checklist-Youth Version (Forth and Kosson 2003) is a notable exception.

3 Because it can be argued that categorizing data will result in a loss of power, we also performed regressions analyses using continuous variables. Yet, these models had severe multicollinearity issues when entering the interaction terms in the analyses.
