# Supplementary material for: Comparing Different Approaches for Subtyping Children with Conduct Problems: Callous-Unemotional Traits Only Versus the Multidimensional Psychopathy Construct
Source: J Psychopathol Behav Assess. 2018 Mar 9;40(1):6–15. doi: 10.1007/s10862-018-9653-y (PMC5860105; doi:10.1007/s10862-018-9653-y)
Supplement: Supplementary file 1 — (DOCX 36 kb) [file 10862_2018_9653_MOESM1_ESM.docx]

Supplementary Material (for review purposes – will be available upon request )

| *Supplement Table 1*  Number (%) of Boys (n = 248) and Girls (n = 284) In Various Groups When Using .75 SD as Cut-Off | | |
| --- | --- | --- |
|  | Boys | Girls |
|  |  |  |
| Control | 174 (70.2) | 211 (74.3) |
| CP Only | 13 (5.2) | 4 (1.4) |
| CU Only | 20 (6.2) | 32 (11.3) |
| Psychopathic Personality | 10 (3.1) | 17 (6.0) |
| CU+CP | 14 (4.4) | 5 (1.8) |
| Psychopathic Personality+CP | 17 (5.3) | 15 (5.3) |
| *Note*. When using the .5 SD as cut-off score, the total number of boys and girls is different from the total number of boys and girls used in the analyses that relied on the .75 SD cut-off score. This is because the number of participants assigned to groups not included in the study (see Method Section) varied as well; CP = conduct problems; CU = callous-unemotional | | |

| Supplement Table 2  Predicting Future and Stable Conduct Problem: Unstandardized Regression Coefficients (B) with 95% Confidence Intervals, and Odds Ratios (OR) With 95% Confidence Intervals | | | | | | | | | | | | |
| --- | --- | --- | --- | --- | --- | --- | --- | --- | --- | --- | --- | --- |
|  | 6-Months Later | | | | 12 Months Later | | | | Stable | | | |
|  | Boys | | Girls | | Boys | | Girls | | Boys | | Girls | |
| **> .5 SD** | B | (95%CI) | B | (95%CI) | B | (95%CI) | B | (95%CI) | OR | (95%CI) | OR | (95%CI) |
| CP Only | 1.52 | (.59; 2.45) | 1.07 | (.01; 2.14) | 1.50 | (.39; 2.60) | .49 | (-.59; 1.56) | 5.52*** | (1.13; 23.11) | 5.08* | (1.16; 22.28) |
| CU Only | -.25 | (-.94; .44) | .32 | (-.33; .97) | -.16 | (-.98; .66) | .15 | (-.50; .81) | >0.00 | na^a^ | 1.69 | (.46; 6.18) |
| PP Only | .66 | (-.20; 1.53) | 1.05 | (.18; 1.92) | .95 | (-.07; 1.97) | 1.06 | (.19; 1.94) | 2.43 | (.50; 11.92) | 5.64** | (1.57; 20.21) |
| CU+CP | .70 | (-.33; 1.73) | 1.72 | (.82; 2.62) | 1.46 | (.24; 2.68) | 1.35 | (.44; 2.25) | 1.40 | (.16; 12.51) | 4.22* | (1.04; 17.22) |
| PP+CP | 1.96 | (1.27; 2.56) | 2.72 | (2.05; 3.39) | 2.70 | (1.88; 3.52) | 3.36 | (2.68; 4.04) | 9.65*** | (3.48; 26.75) | 17.42*** | (6.62; 45.85) |
| **> .75 SD** |  |  |  |  |  |  |  |  |  |  |  |  |
| CP Only | 1.32 | (.40; 2.24) | 1.84 | (.21; 3.47) | 1.29 | (.21; 2.37) | 1.09 | (-.54; 2.72) | 4.04* | (>1.00; 16.30) | 11.03* | (1.39; 87.54) |
| CU Only | -.37 | (-1.12; .39) | .51 | (-.10; 1.11) | -.47 | (-1.36; .42) | .24 | (-.37; .85) | >00 | na^a^ | 1.49 | (.48; 4.68) |
| PP Only | .19 | (-.85; 1.23) | 1.06 | (.25; 1.88) | .55 | (-.67; 1.76) | .87 | (.06; 1.68) | 1.36 | (.16; 11.35) | 3.65* | (1.07; 12.44) |
| CU+CP | .82 | (-.08; 1.72) | 2.01 | (.55; 3.46) | 2.07 | (1.02; 3.12) | .77 | (-.68; 2.23) | .78 | (.09; 6.50) | 8.23* | (1.28; 52.98) |
| PP+CP | 1.50 | (.69; 2.32) | 2.87 | (2.01; 3.72) | 2.21 | (1.26; 3.12) | 4.01 | (3.16; 4.87) | 6.54** | (2.12; 20.17) | 18.86*** | (5.89; 60.39) |
| Note. CP = conduct problems; CU = callous-unemotional; PP = Psychopathic Personality; In all analyses age and parental SES were included as control variables; na = not applicable due to low numbers in the cells.  ^a^ When excluding the CU Only dummy variable from the analyses, results remained substantially similar | | | | | | | | | | | | |
